# Molecular analysis of selected cell cycle regulatory proteins during aerobic and hypoxic maintenance of human ovarian carcinoma cells

**DOI:** 10.1038/sj.bjc.6690615

**Published:** 1999-08

**Authors:** A Krtolica, N A Krucher, J W Ludlow

**Affiliations:** Department of Biochemistry and Biophysics, University of Rochester School of Medicine and Dentistry, Rochester, NY, USA; University of Rochester Cancer Center, 601 Elmwood Avenue, PO Box 704, Rochester, NY 14642, USA

**Keywords:** ovarian cancer, hypoxia, pRB, phosphatase activity, mammalian cell cycle

## Abstract

We have previously reported on the development of an in vitro model system for studying the effect of hypoxia on ovarian carcinoma cell proliferation and invasion (Krtolica and Ludlow, 1996). These data indicate that the cell division cycle is reversibly arrested during the G1 phase. Here, we have continued this study to include the proliferation properties of both aerobic and hypoxic human ovarian carcinoma cells at the molecular level. The growth suppressor product of the retinoblastoma susceptibility gene, pRB, appears to be functional in these cells as determined by SV40 T-antigen binding studies. Additional G1-to-S cell cycle regulatory proteins, cyclins D and E, cyclin-dependent kinases (cdks) 4 and 2, and cdk inhibitors p27 and p18, also appear to be intact based on their apparent molecular weights and cell cycle stage-specific abundance. During hypoxia, there is a decrease in abundance of cyclins D and E, with an increase in p27 abundance. cdk4 activity towards pRB and cdk2 activity towards histone H1 are also decreased. Co-precipitation studies revealed an increased amount of p27 complexing with cyclin E-cdk2 during hypoxia than during aerobic cell growth. In addition, pRB-directed phosphatase activity was found to be greater in hypoxic than aerobic cells. Taken together, a model is suggested to explain hypoxia-induced cell cycle arrest in SKA human ovarian carcinoma cells. © 1999 Cancer Research Campaign


*British Journal of Cancer* (1999) **80**(12), 1875–1883
© 1999 Cancer Research Campaign
Article no. bjoc.1999.0615

### Molecular analysis of selected cell cycle regulatory proteins during aerobic and hypoxic maintenance of human ovarian carcinoma cells

**A Krtolica[1], NA Krucher[2] and JW Ludlow[1,2]**

[1]Department of Biochemistry and Biophysics, University of Rochester School of Medicine and Dentistry, Rochester, NY, USA; [2]University of Rochester Cancer Center, PO Box 704, 601 Elmwood Avenue, Rochester, NY 14642, USA

**Summary** We have previously reported on the development of an in vitro model system for studying the effect of hypoxia on ovarian carcinoma cell proliferation and invasion (Krtolica and Ludlow, 1996). These data indicate that the cell division cycle is reversibly arrested during the G1 phase. Here, we have continued this study to include the proliferation properties of both aerobic and hypoxic human ovarian carcinoma cells at the molecular level. The growth suppressor product of the retinoblastoma susceptibility gene, pRB, appears to be functional in these cells as determined by SV40 T-antigen binding studies. Additional G1-to-S cell cycle regulatory proteins, cyclins D and E, cyclin-dependent kinases (cdks) 4 and 2, and cdk inhibitors p27 and p18, also appear to be intact based on their apparent molecular weights and cell cycle stage-specific abundance. During hypoxia, there is a decrease in abundance of cyclins D and E, with an increase in p27 abundance. cdk4 activity towards pRB and cdk2 activity towards histone H1 are also decreased. Co-precipitation studies revealed an increased amount of p27 complexing with cyclin E-cdk2 during hypoxia than during aerobic cell growth. In addition, pRB-directed phosphatase activity was found to be greater in hypoxic than aerobic cells. Taken together, a model is suggested to explain hypoxia-induced cell cycle arrest in SKA human ovarian carcinoma cells.

**Keywords:** ovarian cancer; hypoxia; pRB; phosphatase activity; mammalian cell cycle

Ovarian cancer is the second most common gynaecological cancer and is the leading cause of death from all gynaecological malignancies. Over 90% of ovarian cancers are believed to result from the malignant transformation of the ovarian surface epithelium. Although the cause of epithelial ovarian carcinoma remains unknown, it has been proposed that the repeated requirement for growth activity in the surface epithelium without long periods of rest somehow contributes to the process of malignant transformation (Fathalla, 1971). The poor prognosis for ovarian epithelial malignancies is most likely related to the extensive dissemination of tumour cells beyond the confines of the ovary at the time of initial diagnosis (American Cancer Society, 1984). After 20 years of intensive research there are still significant gaps in our knowledge concerning ovarian cancer aetiology, development and treatment.

One of the hallmarks of the tumour microenvironment is regional hypoxia occurring because of insufficiencies in the vasculature of the growing neoplasm (Moulder and Rockwell, 1987). In accordance with the need by the cell to decrease energy usage under low oxygen conditions, hypoxia induces cell cycle arrest in many cell types (Heacock and Sutherland, 1990; Amellem and Peterson, 1991; Graeber et al, 1994). We and others (Amellem and Peterson, 1991; Graeber et al, 1994; Krtolica and Ludlow, 1996) have shown that hypoxia induces cells to arrest preferentially in the G1 phase or at the G1/S transition. Results from our laboratory (Ludlow et al, 1993*b*; Krtolica and Ludlow, 1996) as well as others (Amellem et al, 1996) demonstrate that concomitant with this arrest is an accumulation of the hypophosphorylated, growth suppressive form of the retinoblastoma protein, pRB. The pRB protein regulates the cell's decision during G1 to start another round of cell division. Graeber et al (1994) have shown that despite the accumulation of another tumour suppressor gene product, p53, in at least some cell types during hypoxia, p53 does not contribute to the observed G1 arrest. Taken together, these observations are consistent with the hypothesis that G1 arrest in hypoxia may be regulated through activation of the growth suppressive function of pRB.

We have also previously shown that while hypoxic ovarian carcinoma cells undergo reversible G1 cell cycle arrest, they still retain their invasive capability (Krtolica and Ludlow, 1996). It has also been well-established by others that cells under hypoxia often demonstrate resistance to both radiation and chemical therapies designed to halt their growth and proliferation (Wilson et al, 1989; Sutherland, 1990; Hall, 1994). Therefore, it is tempting to speculate that in ovarian malignancies resistant hypoxic cells play a role in maintaining a critical threshold of neoplastic cells capable of repopulating the patient. Hence, identifying the critical biochemical components of this hypoxia-induced growth suppression mechanism will most certainly contribute towards our understanding in the fields of cell cycle control, hypoxia and potentially in experimental therapeutics. Towards this goal, we have continued to study the proliferation properties of both aerobic and hypoxic human ovarian carcinoma cells at the molecular level. The purpose of the study presented here is to assess the integrity

*Received 18 August 1998*
*Revised 8 December 1998*
*Accepted 17 February 1999*

*Correspondence to:* JW Ludlow and complex formation of several key cell cycle regulatory proteins. Since hypoxia reversibly growth arrests SKA cells during the G1 phase of the cell cycle (Krtolica and Ludlow, 1996), we concentrated our investigative efforts on the proteins and their complexes which are known to regulate progression from G1 into S phase: cyclins D and E, the cyclin-dependent kinases (cdk) 4 and 2, the cdk inhibitors (cdki) p18 and p27, and the negative growth regulatory protein pRB. With this information, we propose a model to explain the effects of hypoxia on ovarian cancer cell proliferation.

### MATERIALS AND METHODS

### Cell growth conditions

SKA is a continuous line of ovarian carcinoma cells derived from patient isolates (Rofstad and Sutherland, 1988). SV80 is a transformed human diploid fibroblast cell line which stably expresses SV40 T-antigen (Todaro et al, 1966). These cells were maintained in Dulbecco's modified Eagle's medium (DMEM; Gibco), containing 10% fetal bovine serum at 37°C in a humidified, 5% carbon dioxide-containing atmosphere.

### Flow cytometry and DNA histogram analysis

Aerobic and hypoxic cells were harvested by trypsinization and low speed centrifugation. The resulting cell pellet was then fixed in ice-cold 70% ethanol and stored at 4°C until flow cytometric analysis. Fixed cells (at least $5 \times 10^5$ cells) were then centrifuged (55 ***g***, 10 min), resuspended in 1 ml of phosphate-buffered saline containing 1 mg $ml^{-1}$ RNAase and incubated at room temperature for 30 min. Propidium iodide was added to a final concentration of 10 μg $ml^{-1}$, and flow cytometry was performed on a Profile 1 flow cytometer (Coulter Counter, Coulter Electronics, Inc., Miami, FL, USA). DNA histogram analysis was performed using the MultiCycle AV DNA software package (Phoenix Flow Systems, Inc., San Diego, CA, USA).

### Cell elutriation

Approximately $10^8$ cells were loaded into the separation chamber at a flow rate of 36 ml per min and a rotor speed of 3250 rpm (Beckman J6M centrifuge, JE-6 rotor, Beckman). Samples were collected at a flow rate of 48 ml per min. After collecting the run-off samples, rotor speed was decreased incrementally and 40-ml samples collected at each rotor speed. A total of 28 fractions were collected. The cells contained in these fractions were subsequently counted and sized using a Coulter counter (Model ZM, Coulter Electronics, Inc., Miami, FL, USA) and fractions containing population of cells in the same phase of the cell cycle (same size) were pooled. Cell cycle distribution was confirmed by flow cytometric DNA analysis.

### T-antigen binding and coprecipitation

SKA cells were starved for 30 min in methionine-free medium, labelled with 0.5 mCi $ml^{-1}$ of $[^{35}S]$Met/Cys for 3 h, and lysed in EBC buffer (50 mM Tris–HCl, pH 8.0, 120 mM sodium chloride (NaCl), 0.5% Nonidet P-40) containing 10 μg $ml^{-1}$ of the protease inhibitors aprotinin, leupeptin and phenylmethylsulphonyl fluoride (PMSF). T-antigen containing SV80 cell lysate was then mixed with $[^{35}S]$-labelled SKA lysate at a ratio 1:3 and incubated for 15 min at 4°C prior to immunoprecipitation with T-antigen specific monoclonal antibody (PAB 419; Ludlow et al, 1989). Immunoprecipitates were separated on 7.5% acrylamide gels and visualized by autoradiography.

### Establishing hypoxic cultures

Hypoxic conditions were established by placing cell cultures in sealed chambers maintained at 37°C followed by repeated exchange with 95% $N_2$:5% carbon dioxide over a 2-h period. Oxygen levels were measured in the conditioned medium at the end of each experiment using an oxygen microelectrode (Microelectrodes, Inc., Bedford, NH, USA) and were consistently found to range between 0.5 and 1.5% oxygen, which calculates to 3.8–11.4 mmHg. For comparison, atmospheric oxygen (aerobic conditions) equals 160 mmHg, the partial pressure of oxygen in venous blood is 30–38 mmHg (Siggaard-Anderson et al, 1990), while oxygen levels in tumours and spheroids are 10 mmHg (1.3%) or lower (Sutherland, 1988). From our previous work (Ludlow et al, 1993*b*; Krtolica and Ludlow, 1996; Krtolica et al, 1998), kinetic data on the time course of hypoxia indicated that by 12–18 h the cells had reached maximum growth arrest. The observed cellular effects of hypoxia were not due to depletion of glucose; measurement of glucose levels in conditioned medium from aerobic and hypoxic cell cultures revealed that under all conditions glucose remained above 80% of the amount present in the fresh medium (Sigma Diagnostics Glucose, Procedure No. 510, Sigma, St Louis, MO, USA). There was no induction of cell death under hypoxic conditions as determined by trypan blue exclusion.

### SDS-PAGE and Western blotting

All cells were lysed for 15 min at 4°C in EBC buffer (50 mM Tris–HCl, pH 8.0, 120 mM NaCl, 0.5% Nonidet P-40) containing 10 μg $ml^{-1}$ of the protease inhibitors aprotinin, leupeptin and PMSF. The lysates were cleared by centrifugation at 14 000 ***g*** for 10 min. Electrophoresis was performed in 7.5%, 8%, 10% or 12% sodium dodecyl sulphate (SDS)-polyacrylamide gels (Laemmli, 1970) using 100 μg of total cell protein for each sample lane (Bradford, 1976). After electrophoresis, the proteins were transferred to nitrocellulose paper in buffer containing 25 mM Tris–HCl, 192 mM glycine, 20% v/v methanol, and 0.01% SDS (pH 8.5) (Towbin et al, 1979). Residual protein binding sites on the nitrocellulose were blocked by incubation for 30 min in TBST (25 mM Tris–HCl, pH 8.0, 150 mM NaCl, 0.5% Tween-20) containing 4% non-fat dry milk. Next, the nitrocellulose was incubated in TBST containing 2% non-fat dry milk containing either a 1:100 dilution of antibody to cyclin A (C160; Giordano et al, 1989, 1991), a 1:5 dilution of antibody to cyclin E (HE 12; provided by Ed Harlow and Nick Dyson, Massachusetts General Hospital, Boston, MA, USA), a 1 μg $ml^{-1}$ concentration of cyclin $D_{1,2}$ antibody (06-137; Upstate Biotechnology, Inc. (UBI), Lake Placid, NY, USA), 1 μg $ml^{-1}$ of anti-cdk2 (rabbit polyclonal, 06–505; UBI), 1 μg $ml^{-1}$ anti-cdk4 (SC-260; Santa Cruz Biotechnology, Inc., Santa Cruz, CA, USA), 0.125 μg $ml^{-1}$ of anti-p27 (cat. No. K25020; Transduction Laboratories, Inc., Lexington, KY, USA), or 1 μg $ml^{-1}$ of anti-p18 (cat. No. 06-555; UBI) Following three washes of 10 min each with TBST, the nitrocellulose was probed with horseradish peroxidase-conjugated anti-IgG

 *© 1999 Cancer Research Campaign*

(Promega, Madison, WI, USA) and developed using chemiluminescence detection (Pierce, Rockford, IL, USA) according to the manufacturer's instructions.

### Preparation of [$^{32}$P]pRB substrate and pRB-directed phosphatase activity assays

To assay pRB-directed phosphatase activity, [$^{32}$P]pRB substrate was prepared by immunoprecipitation with monoclonal antibody to pRB, PMG3-245 (PharMingen, San Diego, CA, USA) from [$^{32}$P]-labelled SKA cells as described previously (Nelson et al, 1997*a*, 1997*b*). For enzyme assays, cells were plated at $8 \times 10^5$ cells per 100-mm plate 16–18 h prior to experiment. At the end of the treatment, cells were washed three times with 20 mM imidazole, pH 7.0, 150 mM NaCl and lysed by sonication in the same buffer supplemented with 14 mM 2-mercaptoethanol and the following protease inhibitors: 1 mM benzamidine, 50 μM TPCK, 50 μM TLCK, 50 μM leupeptin and 1 μM pepstatin. Lysates from hypoxic or aerobic cells, normalized for protein content and volume, were incubated with [$^{32}$P]-labelled immunoprecipitated pRB for 40 min at 30°C (assay samples) or mixed immediately with SDS polyacrylamide gel electrophoresis (SDS-PAGE) sample buffer (control samples). Phosphatase reactions were terminated by the addition of SDS-PAGE sample buffer and boiling. Proteins were separated on 8% SDS-polyacrylamide gels. Gels were fixed, dried and activity was measured by assessing incorporation of pRB radiolabel relative to controls and quantified with a phosphorimager and ImageQuant Software (Molecular Dynamics, Sunnyvale, CA, USA). Activity is measured as the percent decrease in pRB band densities in experimental lanes compared to control lanes.

### Immunoprecipitation of cdk4 and cdk2 complexes

Cell lysates were prepared by scraping cells from the plate into ice-cold kinase buffer (20 mM MOPS, pH 7.2, 25 mM β-glycerol phosphate, 5 mM EGTA, 1 mM sodium orthovanadate, 1 mM dithiothreitol (DTT) containing protease inhibitors (10 μg $ml^{-1}$ of aprotinin, leupeptin and PMSF) followed by sonication. Lysates from hypoxic or aerobic cells containing 500 μg of total protein were normalized to the same volume (approximate protein concentration of 1 mg $ml^{-1}$) and following a 1 h pre-clearing with *Staphylococcus* protein A-Sepharose beads, immunoprecipitated for 2 h with primary antibody at 4°C. Next, 50 μl of a 1:1 slurry of protein A-beads in the kinase buffer was added and the precipitates incubated for an additional hour. After three washes with kinase buffer, precipitates were either immediately used for the kinase assay or prepared for SDS-PAGE by the addition of 50 μl of 3 × SDS-PAGE sample buffer (6% SDS, 30% (v/v) glycerol, 0.3 M DTT, 0.36 M Tris–HCl, pH 6.8) and then boiled. The following rabbit polyclonal antibodies were used for these immunoprecipitations: anti-cdk2 (06-505; UBI), anti-cdk4 (Sc-260; Santa Cruz Biotechnology).

### cdk2 kinase assays

Cell lysates were prepared and cdk2 immunoprecipitated as described above. Assays were performed according to manufacturer's instructions (Cyclin-dependent Kinase Assay Kit, 17-137; UBI) in the presence of the following kinase inhibitors: protein kinase C and protein kinase A inhibitor peptides and the calmodulin-dependent kinase inhibitor compound R24570. Reactions were terminated by cooling the mixtures in an ice water bath. The sample was then mixed with SDS-PAGE sample buffer, boiled and proteins separated on 12% SDS-polyacrylamide gels. Gels were fixed, dried and activity was measured by assessing incorporation of radiolabel into histone H1 relative to controls and quantified using a phosphorimager with ImageQuant Software (Molecular Dynamics, Sunnyvale, CA, USA).

### pRB substrate preparation and cdk4 kinase assays

Full-length pRB fused to glutathione-S-transferase (GST-pRB$^{(1–928)}$) was expressed and purified from *Escherichia coli* strain BL21pLys using glutathione-Sepharose beads as described recently (Zarkowska and Mittnacht, 1997). GST-pRB$^{(1–928)}$ was then eluted from the beads using excess glutathione, and estimates of the amount of eluted GST-pRB$^{(1–928)}$ were made by comparing silver-stained band intensities with those of known quantities of bovine serum albumin. Approximately 2 μg of GST-pRB$^{(1–928)}$ was then mixed with cdk4 immunoprecipitates from aerobic and hypoxic-treated cells in kinase buffer (20 mM MOPS, pH 7.2, 25 mM β-glycerol phosphate, 5 mM EGTA, 1 mM sodium orthovanadate, 1 mM DTT) containing protease inhibitors (10 μg $ml^{-1}$ of aprotinin, leupeptin and PMSF). Following the addition of 1 μCi [$^{32}$P]-ATP and incubation at 30°C for 30 min, the reactions were terminated by adding SDS-sample buffer and boiling. Negative control reactions using pre-immune rabbit serum and purified mouse IgG immunoprecipitations revealed undetectable levels of substrate phosphorylation.

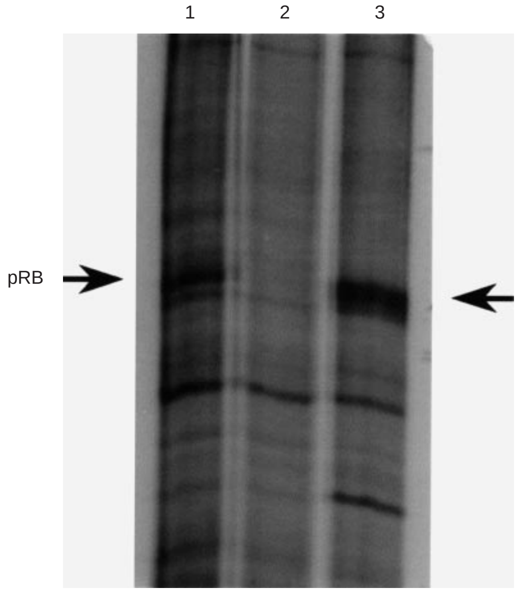

**Figure 1** T-antigen coprecipitation of pRB from SKA cells. [$^{35}$S]-labelled SKA cell lysate was mixed with the non-labelled SV40 T-antigen-containing SV80 cell lysate and immunoprecipitated with T-antigen-specific antibody PAB 419. Samples were analysed using SDS-PAGE and autoradiography. Lanes: 1 – T-antigen immunoprecipitation of radiolabelled SKA cell lysate mixed with T-antigen-containing SV80 cell lysate; 2-immunoprecipitation of radiolabelled SKA cell lysate alone using T-antigen-specific antibody; 3-immunoprecipitation of radiolabelled SKA cell lysate using pRB-specific antibody

© 1999 Cancer Research Campaign

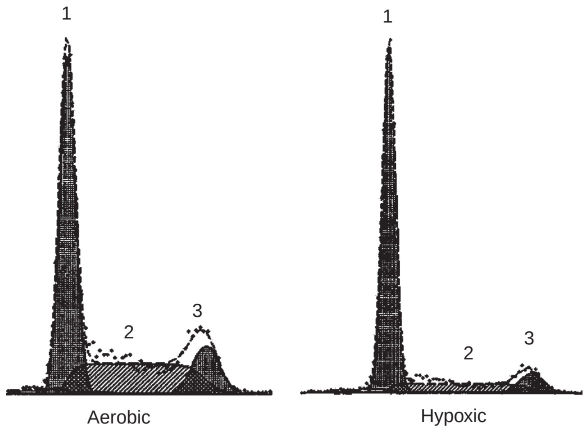

**Figure 2** DNA histogram analysis of aerobic and hypoxic SKA ovarian carcinoma cells. Cell cultures were prepared as aerobic (left panel) or 18-h hypoxic (right panel). Cell cycle distribution was determined by DNA histogram analysis, as described in Materials and Methods. Peaks represent populations of cells in G0/G1, S, and G2/M (peaks 1, 2 and 3 respectively)

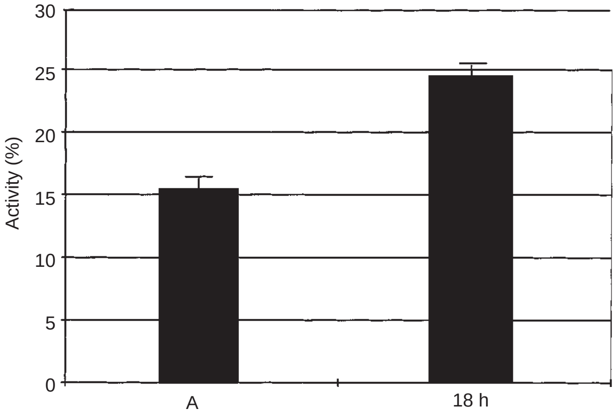

**Figure 3** Percent increase in pRB-directed phosphatase activity in hypoxia relative to aerobic control. Cells were subjected to 18 h of hypoxia (18 h) or maintained under aerobic conditions (A) and after lysis subjected to phosphatase assay as described in Materials and Methods. The data are presented as the average increase in pRB-specific phosphatase activity relative to aerobic controls. Error bars represent standard error of the mean for two samples performed in duplicate

### RESULTS

### pRB from the SKA ovarian carcinoma cell line appears to be wild-type and functional

It has been shown (Horowitz et al, 1989; Ludlow et al, 1989; Bookstein et al, 1990; Kaye et al, 1990) that hypophosphorylated wild-type pRB, but not its mutated inactive forms, binds to SV40 T-antigen. The ability of pRB to act as a growth suppressor directly correlates with its ability to bind T-antigen. Thus, the ability of pRB from particular cells to bind to T-antigen is taken as a positive indicator of its functionality and wild-type status. To test if pRB in SKA cells can bind to T-antigen, extracts were prepared from [$^{35}$S]-labelled SKA cells and mixed in a 1:3 ratio with extracts prepared from T-antigen containing SV80 cells. T-antigen was then immunoprecipitated with specific antibody and the immunoprecipitates separated by SDS-PAGE and analysed by autoradiography for the presence of radiolabelled pRB. As shown in Figure 1, T-antigen immunoprecipitates of these mixed lysates contained radiolabelled pRB which coprecipitated with T-antigen. This result, taken together with reverse transcription polymerase chain reaction (RT-PCR) and single-strand conformation polymorphism (SSCP) analysis which failed to detect aberations in regions of pRB most often found mutated in *RB1* non-functional mutants (Horowitz et al, 1989; Bookstein et al, 1990; Kaye et al, 1990) further supports our view that SKA ovarian carcinoma cells express wild-type, functional, pRB.

### pRB-directed phosphatase activity increases in SKA cells exposed to hypoxia

As reported earlier (Krtolica and Ludlow, 1996) and also illustrated here (Figure 2), flow cytometery of asynchronously growing SKA cells reveals an arrest in G1 or at the G1-S border during hypoxia, as evidenced by a reduction in the S and G2-M peaks of the DNA histogram. Such hypoxia-induced cell cycle arrest is coincident with hypophosphorylation of pRB. One possible explanation for this observation is that the intracellular phosphatase activity directed towards pRB may be increased. Since we have previously identified the pRB-directed phosphatase as belonging to the type I class of serine/threonine protein phosphatases (PP1) and developed an in vitro assay to measure such activity (Ludlow et al, 1993*a*), we tested the hypothesis that PP1 activity towards pRB is increased in SKA cells during hypoxia. As shown in Figure 3, our experiments revealed a 48% increase in pRB-directed phosphatase activity in hypoxic cell lysate relative to the aerobic control. These data support our view that hypoxia-induced hypophosphorylation of pRB, which renders this protein active with respect to growth suppression (Buchkovich et al, 1989; Chen et al, 1989; DeCaprio et al, 1989; Bartek et al, 1996), involves an increase in PP1-mediated pRB dephosphorylation.

### While cyclin $D_{1,2}$ and cdk4 complexes are present in SKA cells during hypoxia, cdk4 activity towards pRB is decreased

Cyclin D–cdk4 and cyclin D–cdk6 activities enable cells to progress through the G1 phase of the cell cycle and are some of the main targets of cell cycle regulation (Resnitzky and Reed, 1995; reviewed by Sherr, 1994). Although there are three types of cyclin D, designated $D_1$, $D_2$ and $D_3$, all are not necessarily found within the same cell type. Our decision to concentrate our efforts on cyclin $D_{1,2}$–cdk4 was based on our ability to unequivocably detect these proteins by Western blotting and also measure specific kinase activity. In addition, cyclin $D_{1,2}$ regulation does not appear to be disrupted in these SKA cells (see Figure 5B). As shown in Figure 4A, in SKA cell lysates the overall level of cyclin $D_{1,2}$ decreases during hypoxia. We did detect somewhat of an increase in cyclin $D_{1,2}$ at the 18-h hypoxic time point relative to the 12-h and 24-h hypoxic time points. Although not rigorously addressed here, this increase may represent a population of S and G2/M cells, which when slowed by hypoxia, eventually reach G1 between 12 and 18 h of hypoxia and then arrest. However, this level was still below that of the aerobic control. cdk4 levels also decrease during hypoxia, suggesting that the expression of these two proteins is down-regulated during hypoxia-induced growth arrest of these

 © 1999 Cancer Research Campaign

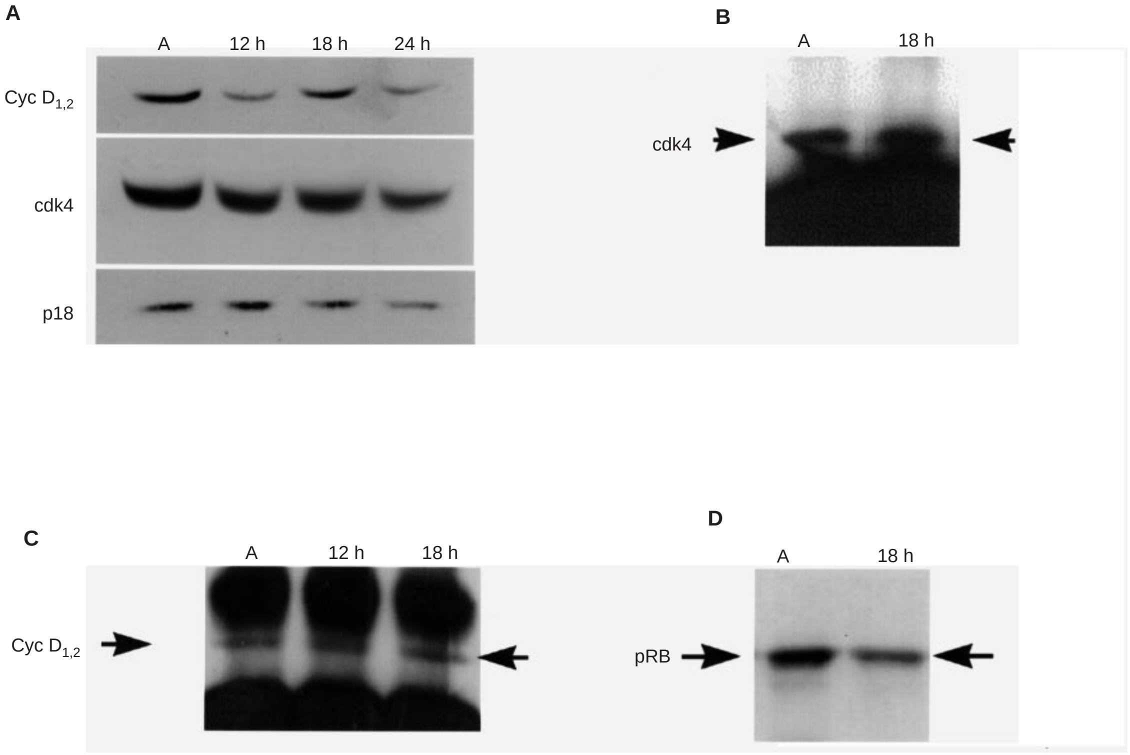

**Figure 4** (**A**) Detection of cyclin $D_{1,2}$, cdk4, and p18 from hypoxic SKA cells. Equal quantities (100 µg) of whole-cell lysates prepared from cells maintained under aerobic conditions (**A**) and 12- (12 h), 18- (18 h) and 24- (24 h) hour hypoxic cells were separated on 12% SDS-gels and subjected to immunoblotting for detection of cyclin $D_{1,2}$ (Cyc $D_{1,2}$), cdk4, and p18 (indicated to the left of each panel) as described in Materials and Methods. (**B**) Immunoprecipitation of cdk4 from aerobic and hypoxic cell lysates. Equal quantities (500 µg) of whole-cell lysates prepared from cells maintained under aerobic conditions (A) and 18-h hypoxic conditions (18 h) were immunoprecipitated using equal quantities of anti-cdk4 (1 µg) and separated on a 12% SDS-gel. Western blots were probed for cdk4, the position of which is indicated by arrows on the left and right of the panel. (**C**) Coprecipitation of cyclin $D_{1,2}$–cdk4 complexes from hypoxic and aerobic cell lysates using cdk4 antibody. Cells were maintained under aerobic conditions (A) or for 12- (12 h) or 18- (18 h) hours in hypoxia, lysed and subjected to immunoprecipitation with cdk4 antibodies as described above and in Materials and Methods. Western blots were probed for cyclin $D_{1,2}$, the position of which is indicated by arrows on the left and right of the panel. (**D**) pRB-directed cdk4 kinase activity. GST-pRB$^{(1–928)}$ bound to glutathione-Sepharose beads was mixed with cdk4 immunoprecipitates from the aerobic and hypoxic cell lysates for the kinase reaction. The proteins were separated on a 6% SDS-gel and phosphorylated pRB visualized by autoradiography ovarian cancer cells. p18, representative of the INK family of cdk inhibitors (p15, p16, p19) which specifically inhibit cyclin D–cdk4 and cyclin D–cdk6 complexes (Guan et al, 1994), also decreases over the time course of hypoxia, albeit less dramatically than cyclin $D_{1,2}$ and cdk4. Experiments using antibody specific for other INK family members yielded inconclusive results, and thus we were unable to accurately determine their abundance in these cells.

Immunoprecipitation studies using cdk4-specific antibody, in which comparable amounts of cdk4 were immunocomplexed (Figure 4B) reveal comparable amounts of cyclin D coprecipitating with cdk4 during both aerobic and hypoxic conditions (Figure 4C). Using cdk4 immunoprecipitates, activity assays towards pRB were also performed. Kinase activity towards pRB is reduced approximately 30% in cdk4 immunoprecipitates from hypoxic cells (Figure 4D). Unfortunately, we were not able to definitively determine the presence of p18 or other cdk inhibitors in these immunoprecipitates. Nonetheless, we conclude from these data that cyclin $D_{1,2}$ from the SKA ovarian carcinoma cell line is capable of forming an active complex with cdk4 during aerobic as well as during hypoxic conditions, although the activity towards pRB is is decreased in hypoxic cells. Taken together, these data suggest that G1 progression during hypoxia is not inhibited by the absence of cyclin $D_{1,2}$–cdk4 complexes, but by the combined effect of a lowering in abundance of the individual components of this complex and a reduction in the catalytic activity of this complex, perhaps resulting from increased binding by one or more types of cdk inhibitors to the cyclin $D_{1,2}$–cdk4 complex during hypoxia.

### Cyclin $D_{1,2}$, cyclin E, cyclin A and p27 protein during SKA cell cycle follow the predicted pattern of expression

Cyclin $D_{1,2}$ can be found in varying concentrations throughout the cell cycle, although there is a slight increase in abundance during middle to late G1. Cyclin E and cyclin A are cdk2-positive regulatory subunits whose amounts vary during the cell cycle and control the availability of the active cdk2 complexes in the cell. Cyclin E–cdk2 activity is necessary for G1 to S transition, while progression through S phase is enabled by cyclin A–cdk2 complexes. p27 is a cdk inhibitor which binds to cdk2 complexes and inhibits their activity. p27 concentration also varies during the cell cycle. To establish if these cell cycle regulatory elements are properly controlled during normal SKA cell cycling, we subjected SKA cells to centrifugal elutriation. Separated populations of the cells in the various phases of the undisrupted cell cycle (Figure 5A) were lysed and used for immunoblot analysis with cyclin $D_{1,2}$ cyclin E, cyclin A and p27 antibodies (Figure 5B)

*© 1999 Cancer Research Campaign*

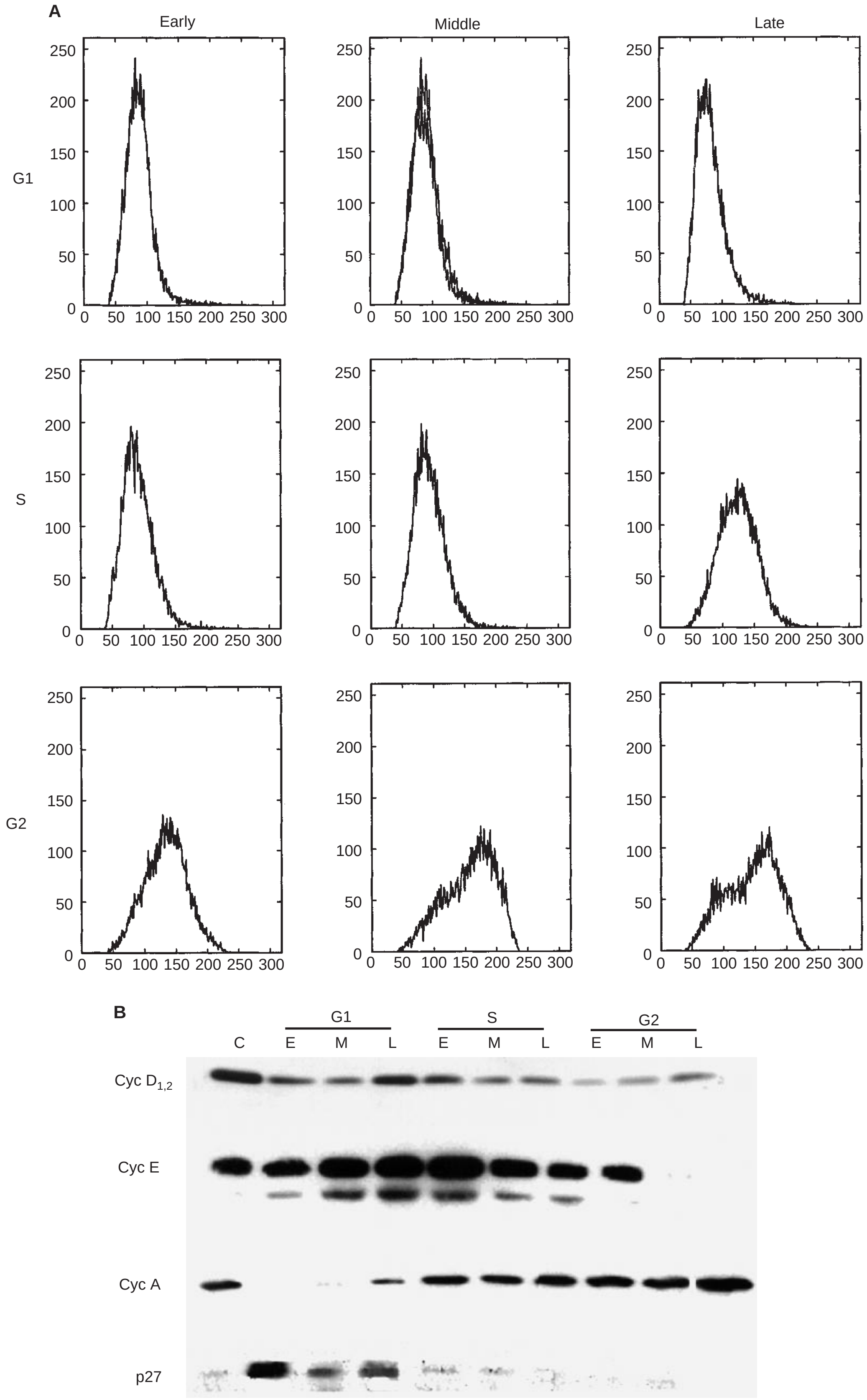

**Figure 5** Distribution of cyclin D, cyclin E, cyclin A and p27 during SKA cell cycle. SKA cells were subjected to centrifugal elutriation. Cells were separated into early (E), mid (M) and late (L) G1, S and G2 populations (panel **A**), lysed, normalized for protein, and used for immunoblot analysis (panel **B**) with cyclin $D_{1,2}$, cyclin E, cyclin A and p27 antibodies (indicated to the left of each panel). C – asynchronous control.

 *© 1999 Cancer Research Campaign*

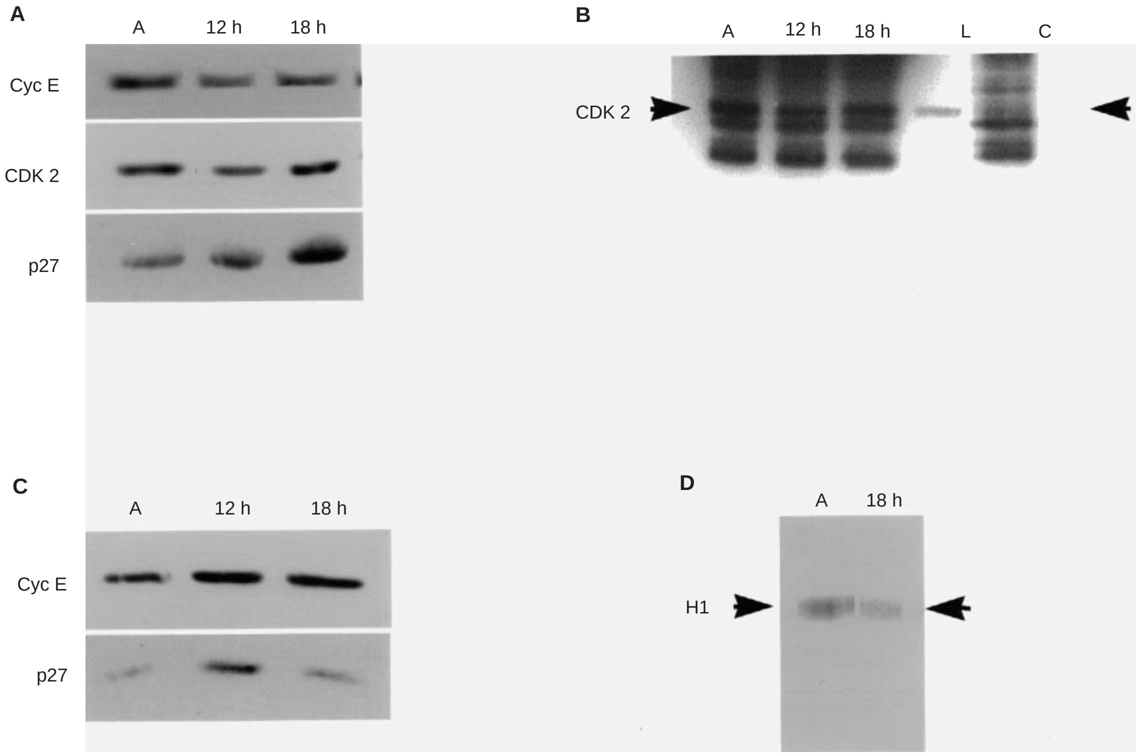

**Figure 6 (A)** Immunoblot analysis of cyclin E, cdk2 and p27 abundance in SKA cell lysates exposed to hypoxia. Equal quantities (100 μg) of whole-cell lysates prepared from cells maintained under aerobic conditions (A), 12- (12 h) and 18- (18 h) hour hypoxic cells were separated on 12% SDS-gels and subjected to immunoblotting for detection of cyclin E (Cyc E), cdk2, and p27 (indicated to the left of each panel) as described in Materials and Methods. (**B**) Immunoprecipitation of cdk2 from aerobic and hypoxic cell lysates. Equal quantities (500 μg) of whole-cell lysates prepared from cells maintained under aerobic conditions (A), 12- (12 H), 18- (18 H) and 24- (24 H) hour hypoxic conditions were immunoprecipitated using equal quantities of anti-cdk2 (1 μg) and separated on a 12% SDS-gel. Western blots were probed for cdk2, the position of which is indicated by arrows on the left and right of the panel. Fifty micrograms of whole-cell lysate (L) and precipitation by normal rabbit serum and protein A beads (C) served as the positive and negative controls respectively. (**C**) Coprecipitation of cyclin E–p27 complexes from hypoxic and aerobic cell lysates using cdk2 antibody. Cells were maintained under aerobic conditions (A) or for 12- (12 h) or 18- (18 h) hours in hypoxia, lysed and subjected to immunoprecipitation with cdk2 antibodies as described above and in Materials and Methods. Western blots were probed for cyclin E and p27 as indicated to the left of the panel. (**D**) Histone H1-directed cdk activity in immunoprecipitates. Immunoprecipitations with cdk2 antibodies and kinase assays were performed as described above and in Materials and Methods. [$^{32}$P]-labelled phosphorylated histone H1 was visualized by autoradiography.

The immunoblot analyses indicate that cyclin $D_{1,2}$ can be found in varying concentrations throughout the cell cycle, with an observable increase during middle to late G1. Cyclin E abundance increases in mid G1 and reaches a peak around the G1/S transition. This is followed by a decrease in mid S phase and its complete disappearance by early G2. The pattern of cyclin E expression during SKA cell cycle is in accordance with reported data which indicate that cyclin E associates with cdk2 in late G1 and that cyclin E associated cdk2 activity is necessary for the G1 to S transition (reviewed by Heichman and Roberts, 1994). Therefore, we concluded that cyclin $D_{1,2}$ and cyclin E regulation does not seem to be disrupted in SKA cells.

Cyclin A complexes with, and regulates the activity of, two cdk catalytic subunits. During late G1 and early S phase cyclin A replaces cyclin E in the cdk2 complexes. It remains bound to cdk2 throughout the S phase and is necessary for S phase progression and entry into G2. In the mid to late G2, cyclin A concentration increases and it binds to cdk1. Together with cyclin B–cdk1 (often referred to as 'maturation factor') activity, cyclin A–cdk1 activity is necessary for the onset of mitosis. Our results indicate that cyclin A abundance during the SKA cell cycle follows the expected pattern of cyclin A expression: it starts to emerge during the G1 to S transition, and increases steadily throughout S and G2 phases. Cyclin A reaches its peak in abundance at the G2 to M transition. This suggests that cyclin A expression does not appear to be altered in the SKA ovarian carcinoma cell line and follows the same pattern as in non-transformed cells. Finally, centrifugal elutriation in conjunction with immunoblot analysis also revealed the expected pattern of p27 expression in SKA; p27 is most abundant in the G1 phase.

### Increased abundance of p27 and cyclin E–cdk2–p27 complexes during hypoxia

Since hypoxic SKA cells appear to be reversibly growth arrested during G1 (Krtolica and Ludlow, 1996), we decided to further focus our attention on the key cdk which regulates transition from G1 into S at the G1/S boundary; namely cyclin E–cdk2 complex. As shown in Figure 6, little change in cdk2 abundance is observed during the time course of hypoxia, while there is a significant increase in p27 and overall decrease in cyclin E abundance compared to the aerobic control. Immunoprecipitation studies using cdk2-specific antibodies, in which comparable amounts of cdk2 can be immunocomplexed from aerobic and hypoxic cell lysate (Figure 6B) reveal that more p27 can be found in cyclin E–cdk2 complexes during hypoxia, especially at the 12 h time

© 1999 Cancer Research Campaign point, than during aerobic conditions (Figure 6C). cdk2 activity towards histone H1 is also reduced approximately 33% in hypoxic cell lysates (Figure 6D), perhaps resulting from this increase in p27 binding to cyclin E–cdk2 complexes.

### DISCUSSION

We have previously shown that ovarian carcinoma cell lines reversibly growth arrest during hypoxia in the G1 phase of the cell cycle and that this arrest is concomitant with accumulation of pRB in its hypophosphorylated state (Krtolica and Ludlow, 1996). In this study we have assessed the functionality of some of the key regulators of the cell cycle in SKA ovarian carcinoma cells and continued our elucidation of the molecular mechanism involved in hypoxia-induced arrest.

Using T-antigen binding, we have demonstrated that pRB is functional in SKA cells. This is in accordance with our hypothesis that activation of pRB may play an important role in hypoxia-induced cell cycle arrest in the SKA cell line. This hypothesis was further supported by the pRB-directed phosphatase assays which revealed that during hypoxia SKA cells increase their pRB-directed phosphatase activity, thus contributing towards maintaining pRB in the growth suppressive, hypophosphorylated state. In addition, immunoblotting detected a decrease in both cyclin $D_{1,2}$ and cdk4 proteins during hypoxia, which implies that the abundance of cyclin $D_{1,2}$–cdk4 complexes should also be reduced. Since the limiting component for active cdk4 complexes is the cyclin D subunit, one may also infer from these data that cdk4 directed kinase activity, for which pRB is the principle substrate, is also decreased during hypoxia. Indeed, our in vitro assay did detect such a reduction. Thus, synergy between increased pRB-directed phosphatase activity and decreased pRB-directed kinase activity may be the mechanism behind the observed hypoxia-induced increase in hypophosphorylated pRB.

In a similar vein, the limiting components for active cdk2 complexes are cyclins E and A. Like cyclin D, both cyclin A (Krtolica and Ludlow, 1996) and cyclin E levels are significantly decreased during hypoxia. In addition, there is an increase in p27 abundance with the onset of hypoxia which results in a higher association of p27 with cdk2 complexes. This suggests a model of hypoxia-induced arrest in which cdk4 activity is lower due to decreased cyclin D and cdk4 abundance, while cdk2 activity is perhaps inhibited through increased binding of p27 and a decrease in availability of the positive regulatory subunits, cyclin E and cyclin A. Since cyclin E–cdk2 and cyclin A–cdk2 complexes are also capable of phosphorylating pRB, down-regulation of their activities during hypoxia is also in accordance with the observed increase in hypophosphorylated pRB during hypoxia, and provides further support for a possible synergistic mechanism between pRB-directed phosphatase and kinase activities to maintain pRB in the hypophosphorylated growth suppressive form upon hypoxic exposure of ovarian carcinoma cells.

These data, taken together with reported inhibition of pRB-directed kinase activity resulting from decrease in G1 cyclin availability and increase in p27–cdk2 association in hypoxic CV-1P cells (Krtolica et al, 1998), has led us to propose the following mechanism of hypoxia-induced growth arrest for these ovarian carcinoma cells. Hypoxia-induced cell cycle arrest prevents cells from starting the cell division cycle, an energetically expensive process, under conditions when energy is limiting. Upon cell exposure to hypoxia, the abundance of cdk inhibitor p27 is elevated and, as a consequence, more p27 is available to bind cyclin–cdk2 complexes, resulting in the decrease in cdk2 kinase activity. At the same time, there is a decrease in the cyclin D and cdk4 amounts leading to a decrease in cdk4 activity. Simultaneous with inhibition of the kinase activity is an increase in pRB-directed phosphatase activity. The concerted action of cdk inhibition and increased pRB dephosphorylation leads to the net accumulation of pRB in its active, hypophosphorylated form. This results in G1 growth arrest through repression of early S phase gene transcription and consequent inhibition of DNA synthesis.

There seems to be a strong similarity between the mechanisms involved in hypoxia induced growth arrest in non-transformed monkey kidney CV-1P cells (Ludlow et al, 1993*b*; Krtolica et al, 1998) and the SKA ovarian carcinoma cell line (Krtolica and Ludlow, 1996). These similarities suggest that our proposed model for hypoxia-induced growth arrest may represent one of the common pathways utilized by multiple cell types in response to hypoxia and does not seem to be connected to the cancer cell phenotype per se. However, we cannot at this time exclude the possibility that this pathway is limited to immortalized cells, since all of the cell types used thus far for our studies belong to this category.

In closing, the results of the experiments described above and those of our previous reports (Ludlow et al, 1993*b*; Krtolica and Ludlow, 1996; Krtolica et al, 1998) lead us to conclude that several key cell cycle regulatory elements: pRB, cyclins D, E, and A, cdk inhibitors p27 and p18, and cdk4 and 2 are present, functional, and capable of forming the appropriate complexes with one another, in the SKA ovarian carcinoma cell line. Our data also suggest that the decrease in cyclin D and cdk4 abundance may modulate cdk4 activity during hypoxia, that increased p27 binding to cyclin E–cdk2 complexes may inhibit cdk2 kinase activity, and together with the observed increase in PP1 phosphatase activity contribute towards maintaining pRB in its growth suppressive, hypophosphorylated state during hypoxia. The next challenge is to formally test the proposed model by molecularly dissecting the significance of each component in the model. In this regard, we have recently reported on a bioreactive protein capable of overriding hypoxia-induced cell cycle arrest of SKA cells (Krucher et al, 1998). When overriding hypoxia-induced cell cycle arrest, we can observe concomitant reversal of several key molecular characteristics reported here in response to hypoxia (i.e. increase in pRB hyperphosphorylation, decrease in p27 abundance) further supporting integral involvement of these proteins in the mechanism of hypoxia-induced cell cycle arrest. Future reports will include these findings.

### ACKNOWLEDGEMENTS

This work was supported by Research Project Grant # 98-108 from the American Cancer Society, The Sally Edelman & Harry Gardner Cancer Research Foundation, University of Rochester Cancer Center Discovery Fund (awarded to JWL) and Cancer Center Core Grant CA11198. NAK is supported by National Institutes of Health Cancer Research Training Grant T32 CA09363.

### REFERENCES

Amellem O and Pettersen EO (1991) Cell inactivation and cell cycle inhibition as induced by extreme hypoxia: the possible role of cell cycle arrest as a protection against hypoxia-induced lethal damage. *Cell Prolif* **24**: 127–141

 *© 1999 Cancer Research Campaign*

Amellem O, Stokke T, Sandvik JA and Pettersen EO (1996) The retinoblastoma gene product is reversibly dephosphorylated and bound in the nucleus in S and G2 phases during hypoxic stress. *Exp Cell Res* **227**: 106–115

Bartek J, Bartkova J and Lukas J (1996) The retinoblastoma protein pathway and the restriction point. *Curr Opin Cell Biol* **8**: 805–814

Bookstein R, Shew JY, Chen PL, Scully P and Lee WH (1990) Suppression of tumorigenicity of human prostate carcinoma cells by replacing a mutated RB gene. *Science* **247**: 712–715

Bradford MM (1976) A rapid and sensitive method for quantitation of microgram quantities of protein utilizing the principle of protein-dye binding. *Anal Biochem* **72**: 248–254

Buchkovich K, Duffy LA and Harlow E (1989) The retinoblastoma protein is phosphorylated during specific phases of the cell cycle. *Cell* **58**: 1097–1105

American Cancer Society (1984) *Cancer Facts and Figures*. Am Cancer Society: New York

Chen PL, Scully P, Shew JY, Wang JYJ and Lee WH (1989) Phosphorylation of the retinoblastoma gene product is modulated during the cell cycle and cellular differentiation. *Cell* **58**: 1193–1198

DeCaprio JA, Ludlow JW, Lynch D, Furukawa Y, Griffin J, Piwnica-Worms H, Huang CM and Livingston DM (1989) The product of the retinoblastoma susceptibility gene has properties of a cell cycle regulatory element. *Cell* **58**: 1085–1095

Fathalla MF (1971) Incessant ovulation: a factor in ovarian neoplasia? *Lancet* **2**: 163

Giordano A, Whyte P, Harlow E, Franza Br Jr, Beach D and Draetta G (1989) A 60 kd cdc2-associated polypeptide complexes with the E1A proteins in adenovirus-infected cells. *Cell* **58**: 981–990

Giordano A, McCall C, Whyte P and Franza BR Jr (1991) Human cyclin A and the retinoblastoma protein interact with similar but distinguishable sequences in the adenovirus E1A gene product. *Oncogene* **6**: 481–485

Guan KL, Jenkins CW, Li Y, Nichols MA, Wu X, O'Keefe CL, Matera AG and Xiong Y (1994) Growth suppression by p18, a p16INK4/MTS1- and p14INK4B/MTS2-related CDK6 inhibitor, correlates with wild-type pRb function. *Genes & Develop* **8**: 2939–2952

Graeber TG, Peterson JF, Tsai M, Monica K, Fornace AJ Jr and Giaccia AJ (1994) Hypoxia induces accumulation of p53 protein, but activation of G1-phase check point by low-oxygen conditions is independent of p53 status. *Mol Cell Biol* **14**: 6264–6277

Hall EJ (1994) The oxygen effect and reoxygenation. In: *Radiobiology for the Radiologist*, 4th edition, pp. 133–152. JB Lippincott: Philadelphia

Heacock CS and Sutherland RM (1990) Enhanced synthesis of stress proteins caused by hypoxia and relation to altered cell growth and metabolism. *Br J Cancer* **62**: 217–225

Heichman KA and Roberts JM (1994) Rules to replicate by. *Cell* **79**: 557–562

Horowitz JM, Yandell DW, Park SH, Canning S, Whyte P, Buchkovich K, Harlow E, Weinberg RA and Dryja TP (1989) Point mutational inactivation of the retinoblastoma antioncogene. *Science* **243**: 937–940

Kaye FJ, Kratzke RA, Gerster JL and Horowitz JM (1990) A single amino acid substitution results in a retinoblastoma protein defective in phosphorylation and oncoprotein binding. *Proc Natl Acad Sci USA* **87**: 6922–6926

Krtolica A and Ludlow JW (1996) Hypoxia arrests ovarian carcinoma cell cycle progression, but invasion is unaffected. *Cancer Res* **56**: 1168–1173

Krtolica A, Krucher NA and Ludlow JW (1998) Hypoxia-induced pRB hypophosphorylation results from downregulation of CDK and upregulation of PP1 activities. *Oncogene* **17**: 2295–2304

Krucher NA, Krtolica A, Lincoln J, Khan SA, Rodriguez-Rodriguez L and Ludlow JW (1998) Mitogenic activity of steroidogenesis-inducing protein (SIP) during hypoxic stress of human ovarian carcinoma cells. *Cancer Letters* **133**: 205–214

Laemmli UK (1970) Cleavage of structural proteins during the assembly of the head of bacteriophage T4. *Nature* **227**: 680–685

Ludlow JW, DeCaprio JA, Huang CM, Lee WH, Paucha E and Livingston DM (1989) SV40 large T antigen binds preferentially to an underphosphorylated member of the retinoblastoma susceptibility gene product family. *Cell* **56**: 57–65

Ludlow JW, Glendening CL, Livingston DM and DeCaprio JA (1993a) Specific enzymatic dephosphorylation of the retinoblastoma protein. *Mol Cell Biol* **13**: 367–372

Ludlow JW, Howell RL and Smith HC (1993b) Hypoxic stress induces reversible hypophosphorylation of pRB and reduction in cyclin A abundance independent of cell cycle progression. *Oncogene* **8**: 331–339

Moulder JE and Rockwell S (1987) Tumor hypoxia: its impact on cancer therapy. *Cancer Metastasis Rev* **5**: 313–341

Nelson DA and Ludlow JW (1997a) Characterization of the mitotic phase pRB-directed protein phosphatase activity. *Oncogene* **14**: 2407–2415

Nelson DA, Krucher NA and Ludlow JW (1997b) High molecular weight protein phosphatase type 1 dephosphorylates the retinoblastoma protein. *J Biol Chem* **272**: 4528–4535

Resnitzky D and Reed SI (1995) Different roles for cyclin D1 and E in regulation of the G1-to-S transition. *Mol Cell Biol* **15**: 3463–3469

Rofstad EK and Sutherland RM (1988) Radiation sensitivity of human ovarian carcinoma cell lines in vitro: effects of growth factors and hormones, basement membrane, and intercellular contact. *Int J Radiation Oncology Biol Phys* **15**: 921–929

Sherr CJ (1944) G1 phase progression: cycling on cue. *Cell* **79**: 551–555

Siggaard-Anderson O, Gothgen IH, Wimberley PD and Fogh-Anderson N (1990) The oxygen status of the arterial blood revised: relevant oxygen parameters for monitoring the arterial oxygen availability. *Scand J Clin Invest* **203**: 17–28

Sutherland RM (1988) Cell and environment interactions in tumor microregions: the multicellular spheroid model. *Science* **240**: 177–184

Sutherland RM (1990) Induction of stress proteins and drug resistance in hypoxia and applications of magnetic resonance spectroscopy and cryospectrophotometry for detecting hypoxia in tumors. In: *Selective Activation of Drugs by Redox Processes*, Adams GE (eds), pp. 97–111. Plenum Press: New York

Todaro GJ, Green H and Swift MR (1966) Susceptibility of human diploid fibroblast strains to transformation by Sv40 virus. *Science* **153**: 1252–1254

Towbin H, Staehelin T and Gordon J (1979) Electrophoretic transfer of proteins from polyacrylamide gels to nitrocellulose sheet: procedure and some applications. *Proc Natl Acad Sci USA* **76**: 4350–4354

Wilson RE, Keng PC and Sutherland RM (1989) Drug resistance in Chinese hamster ovary cells during recovery from severe hypoxia. *J Natl Cancer Inst* **81**: 1235–1240

Zarkowska T and Mittnacht S (1997) Differential phosphorylation of the retinoblastoma protein by $G_1$/S cyclin-dependent kinases. *J Biol Chem* **19**: 12738–12746

© 1999 Cancer Research Campaign 
